# Factors associated with foreign body infection in methicillin-resistant *Staphylococcus aureus* bacteremia

**DOI:** 10.3389/fimmu.2024.1335867

**Published:** 2024-02-16

**Authors:** Kevin Bouiller, Natasia F. Jacko, Margot J. Shumaker, Brooke M. Talbot, Timothy D. Read, Michael Z. David

**Affiliations:** ^1^ Division of Infectious Diseases, Department of Medicine, University of Pennsylvania, Philadelphia, PA, United States; ^2^ Université de Franche-Comté, CHU Besançon, UMR-CNRS 6249 Chrono-environnement, Department of Infectious and Tropical Diseases, Besançon, France; ^3^ Division of Infectious Diseases, Department of Medicine, Emory University School of Medicine, Atlanta, GA, United States

**Keywords:** *Staphylococcus aureus*, biofilm, foreign bodies, methicillin resistance, bacteremia

## Abstract

**Background:**

We aimed to compare patient characteristics, MRSA sequence types, and biofilm production of MRSA strains that did and did not cause a foreign body infection in patients with MRSA bloodstream infections (BSI)

**Methods:**

All adult patients with MRSA BSI hospitalized in two hospitals were identified by clinical microbiology laboratory surveillance. Only patients who had at least one implanted foreign body during the episode of BSI were included.

**Results:**

In July 2018 - March 2022, of 423 patients identified with MRSA BSI, 118 (28%) had ≥1 foreign body. Among them, 51 (43%) had one or more foreign body infections. In multivariable analysis, factors associated with foreign body infection were history of MRSA infection in the last year (OR=4.7 [1.4-15.5], p=0.012) community-associated BSI (OR=68.1 [4.2-1114.3], p=0.003); surgical site infection as source of infection (OR=11.8 [2-70.4], p=0.007); presence of more than one foreign body (OR=3.4 [1.1-10.7], p=0.033); interval between foreign body implantation and infection <18 months (OR=3.3 [1.1-10], p=0.031); and positive blood culture ≥48h (OR=16.7 [4.3-65.7], p<0.001). The most prevalent sequence type was ST8 (39%), followed by ST5 (29%), and ST105 (20%) with no significant difference between patients with or without foreign body infection. Only 39% of MRSA isolates formed a moderate/strong biofilm. No significant difference was observed between patients with foreign body infection and those without foreign body infection. In multivariable analysis, subjects infected with a MRSA isolate producing moderate/strong *in vitro* biofilm were more likely to have a history of MRSA infection in the last year (OR=3.41 [1.23-9.43]), interval between foreign body implantation and MRSA BSI <18 months (OR=3.1 [1.05-9.2]) and ST8 (OR=10.64 [2-57.3]).

**Conclusion:**

Most factors associated with foreign body infection in MRSA BSI were also characteristic of persistent infections. Biofilm-forming isolates were not associated with a higher risk of foreign-body infection but appeared to be associated with MRSA genetic lineage, especially ST8.

## 1 Introduction

The implantation of foreign bodies in human medicine has increased over time related to the aging population and the development of new materials, new surgical methods, and new indications (e.g., transcatheter valve replacement) (1, 2). Foreign body infections pose a significant human and economic burden (3, 4). *Staphylococcus aureus* is one of the most common bacterial species causing foreign body infections (1, 5–8). An accurate and timely diagnosis of foreign body infection is necessary for optimal surgical and antibiotic treatment. One of the major virulence factors contributing to *S. aureus* foreign body infection is the ability of some strains to form a biofilm, cause persistent infection, and develop resistance to antibiotics. Also, biofilms can enable infecting bacteria to escape human immune responses (9–11). In the event of an infection, foreign bodies usually need to be removed because antibiotics are often ineffective at killing bacteria within biofilms. Furthermore, antibiotic resistance in methicillin-resistant *S. aureus* (MRSA) limits antibiotic choice for treatment. However, the role of the intrinsic biofilm-forming characteristics of *S. aureus* strains associated with foreign body infections has been little studied. The aim of this study was to compare patient characteristics, MRSA sequence types, and biofilm production of MRSA strains that did and did not cause a foreign body infection in patients with MRSA bloodstream infections (BSIs).

## 2 Materials and methods

### 2.1 Study population

All adult patients with MRSA bacteremia hospitalized at two hospitals (Hospital of the University of Pennsylvania, and Penn Presbyterian Medical Center) were identified by clinical microbiology laboratory surveillance between July 2018 and March 2022. Only patients who had at least one implanted foreign body during the episode of BSI were included. Patients who died within 48h of hospital admission and patients receiving end-of-life care without specific directed management of MRSA BSI were excluded. Clinical data were retrospectively abstracted from the electronic medical record on a standardized case report form and entered into the online secure database REDCap (Research Electronic Data Capture), including demographics; medical history; history of MRSA infection within the last year; site of acquisition of MRSA BSI; number of days of positive blood cultures; delay between foreign body insertion and infection; initial source of BSI; management and in-hospital mortality; Acute Physiology and Chronic Health Evaluation (APACHE) II score, calculated on the day of the index positive blood culture; and the Pitt bacteremia score.

### 2.2 Definitions

Foreign bodies included pacemakers (PM), implantable cardiac defibrillators (ICD), cardiac resynchronization therapy (CRT) devices, left ventricular assistance devices (LVAD), prosthetic heart valves, prosthetic joints, endovascular implants and bone implants other than those related to prosthetic joints. BSI cases were categorized either as community-associated (CA), community-onset, healthcare–associated (HACO), or healthcare-associated (HA) infection as previously defined (12). Infective endocarditis was diagnosed according to the 2023 modified Duke criteria (13). Cardiac implantable electronic device infection was based on the 2020 international consensus (1). Left ventricular assist device infection related blood stream infection was defined as previously (14). Because no standard diagnostic criteria for osteosynthesis orthopedic infection existed, criteria established for PJIs were used (15). Endovascular implant infection was defined by the MAGIC consensus criteria (16).

### 2.3 Follow-up

Subjects with a foreign body infection were followed to the end of hospitalization or death, and subjects without a foreign body infection at the time of hospitalization were followed to the last medical follow-up visit during the study period.

### 2.4 *In vitro* mature biofilm formation

Biofilm formation was assessed using a crystal violet staining assay in a 96 well-plate, as previously described (17) (see **Supplementary data**). The interpretation of biofilm production was performed according to the criteria of Stepanovic et al. (17). The average optical density (OD) value of all tested strains and negative controls was calculated. Cut-off OD (ODc) was defined as three standard deviations above the mean OD of the negative control. Strains were interpreted as follows: no biofilm producer, OD≤ODc; weak biofilm producer, ODc < OD ≤ 2 × ODc; moderate biofilm producer, 2 × ODc < OD ≤ 4 × ODc; and strong biofilm producer, 4 × ODc < OD.

### 2.5 Molecular typing

Genomic DNA extractions were performed using a commercial kit (Qiagen). Library preparation and whole genome sequencing were performed by the Penn/Children’s Hospital of Pennsylvania Microbiome Center using Illumina MiSeq or Hiseq platforms with paired-end 150 bp reads. MLST types were identified using the Bactopia pipeline (v 1.6.5) (18). PVL genes were identified using the Bactopia subworkflow for ARIBA (v 2.14.6; https://www-microbiologyresearch-org.proxy.library.emory.edu/content/journal/mgen/10.1099/mgen.0.000131) to detect *LukF* and *LukS* genes in assembled genomes. Reference sequences were obtained from the Virulence Factor Database.

### 2.6 Statistical methods

Data are reported as medians (interquartile range [IQR]) for continuous variables and counts (percentages) for categorical variables. Comparisons of continuous data were performed using the Student t-test or the Mann-Whitney U test and categorical data were compared using Pearson’s chi-squared test or Fischer’s exact test, as appropriate. For statistical analysis, moderate and strong biofilm producers were compared to non/weak biofilm producers. Factors independently associated with foreign body infection and with isolates with moderate/strong biofilm formation were determined from a multivariable logistic regression model that was built using a backward elimination process. All independent variables with a p-value < 0.10 in univariable analysis were included in the multivariable model. Statistical analyses were performed using SPSS 28.0 (IBM, Armonk, NY, USA).

## 3 Results

### 3.1 Study population

**Figure 1:**
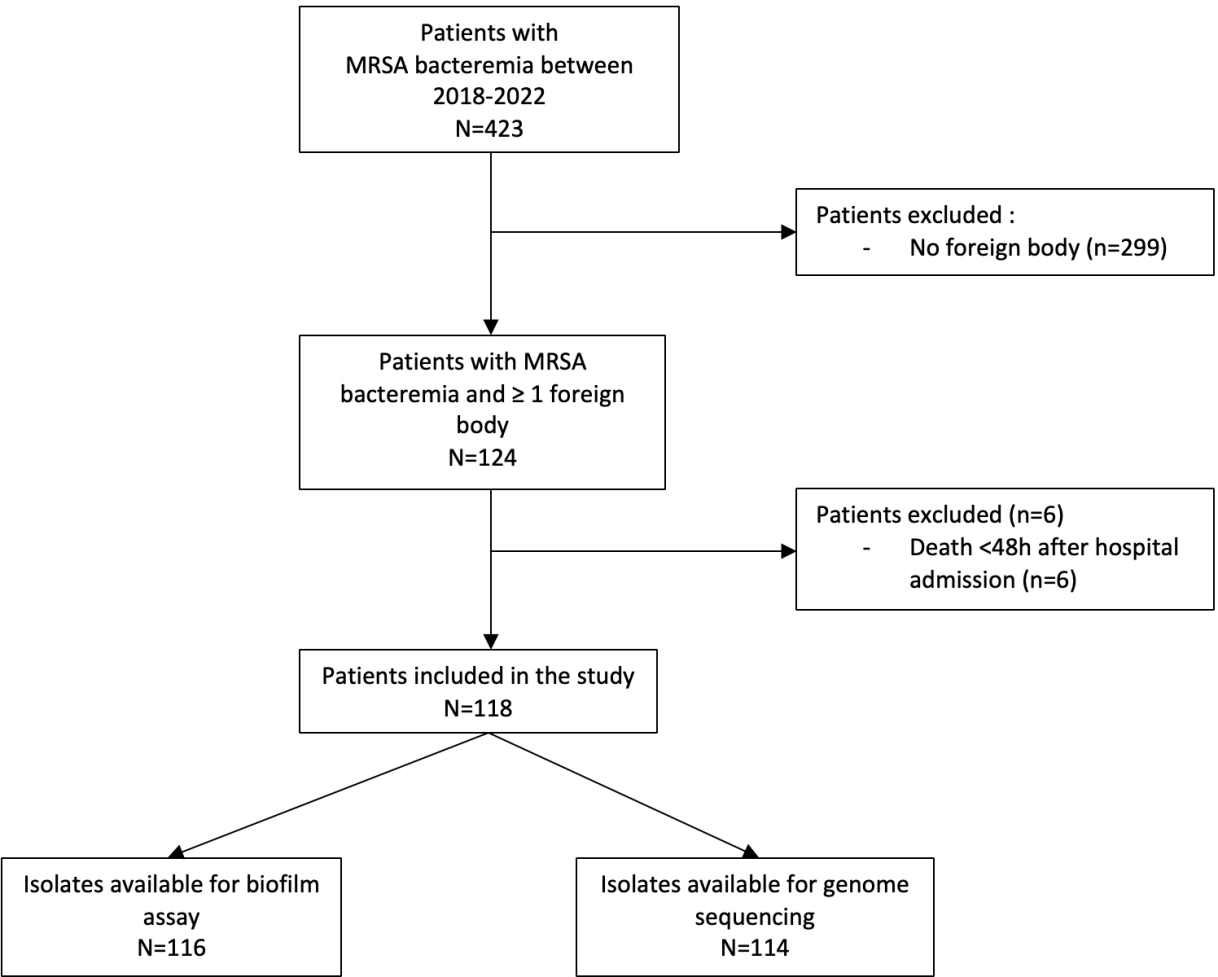
Flow chart.

In July 2018 - March 2022, of 423 patients identified with MRSA BSI, 124 (29%) had a foreign body *in situ*. Among them, 6 were excluded due to death in <48 hours (**Figure 1**). Of 118 eligible subjects, 51 (43%) had one or more foreign body infections: 16 had an intracardiac device infection (13 with PM/ICD infection, 3 with LVAD infection), 13 had a prosthetic heart valve infection, 10 had an endovascular implant infection, 7 had a PJI, and 13 had another type of bone implant infection. Among the 51 subjects with a foreign body infection, 8 had two or more foreign body infections (2 with prosthetic heart valve and PM/ICD infection; 2 with endovascular implant and a prosthetic heart valve infection; 1 with prosthetic heart valve, endovascular implant and PJI; 1 with prosthetic heart valve and PJI; and 1 with PJI and another type of bone implant infection). Moreover, 60/179 (34%) foreign bodies were infected: 13/22 (59%) prosthetic heart valves, 10/20 (50%) endovascular implants, 16/36 (44%) PM/ICD/LVAD (2 patients had 2 intracardiac devices that were not infected), 7/42 (17%) prosthetic joints (11 patients had 2 and 1 had 4 joint prostheses not infected), 13/58 (22%) other types of bone implants (5 patients had 2 and 1 patient had 4 other types of bone implants not infected).

All patients were diagnosed with one or more foreign body infection during the same hospitalization as the MRSA BSI, except for two patients: one subject diagnosed with an endovascular implant infection 2 months after the MRSA BSI diagnosis, and a second subject with PJI 3 months after MRSA BSI. In patients without a foreign body infection, the median follow-up time was 220 days (IQR 43-529). All patients with a prosthetic valve underwent a transthoracic echocardiography (TTE) study, and only 5 subjects did not undergo transesophageal echocardiography (TEE). Among these 5, one had a prosthetic valve infection diagnosed by TTE and had a contraindication to TEE. The other 4 subjects had a repeat TTE not suggestive of infective endocarditis and a follow up time of greater than 300 days without relapse of MRSA BSI or new prosthetic valve surgery. In subjects with an *in situ* cardiac device, all underwent a TTE and 20/34 underwent a TEE. Among 14 subjects who did not undergo TEE, 4 had a diagnosis of cardiac device infection with TTE only and 8 had a second TTE performed. In these 8 patients, blood cultures were positive for only one day.

The median time between foreign body implantation and infection was greater than one year for all types of foreign body except for endovascular implants (0.39 years [IC95%, 0.16-0.61]) and other bone implants (0.98 years [0.00-2.04]) (**Supplementary Figure S1**).

### 3.2 Factors associated with foreign body infection

Patients with foreign body infections were less likely to be black (17/51, 33% vs. 35/67, 52%, p=0.041), less likely to be receiving immunosuppressive therapy (5/51, 9.8% vs. 17/67, 25%, p=0.031), more often had a history of MRSA infection within the last year (18/51, 35%, vs. 13/67, 19%, p=0.052), more often had CA, and less HA BSI (7/51, 13.7%, vs. 1/67, 1.5%, p=0.02 and 6/51, 11.8% vs. 22/67, 32.8%, p<0.01, respectively) (**Table 1**). In multivariable analysis, factors associated with foreign body infection were history of MRSA infection in the last year (OR=4.7 [1.4-15.5], p=0.012), CA-MRSA BSI (OR=68.1 [4.2-1114.3], p=0.003), surgical site infection as source of infection (OR=11.8 [2-70.4], p=0.007), presence of more than one foreign body (OR=3.4 [1.1-10.7], p=0.033), interval between foreign body implantation and infection <18 months (OR=3.3 [1.1-10], p=0.031), and positive blood culture ≥48h (OR=16.7 [4.3-65.7], p<0.001) (**Table 2**).

**Table 1:** Characteristics of 118 patients with MRSA bacteremia and at least one foreign body at time of bacteremia.

Characteristics	Total (n=118)	No foreign body infection (n=67)	Foreign body infection (n=51)	p-value
Age, med [Q25-75]	61.4 [49.4; 69.6]	61.0 [49.5; 71.8]	61.4 [49.8; 68.9]	0.71
Female sex	46 (39)	27 (40)	19 (37)	0.74
Race				0.041
White	48 (41)	26 (39)	22 (43)	
Black	52 (44)	35 (52)	17 (33)	
Other	8 (6.8)	4 (6)	4 (8)	
Unknown	10 (8.5)	2 (3)	8 (16)	
Body mass index, med [Q25-75]	27.3 [24.0; 32.1]	27.2 [23.3; 30.9]	28.8 [25.0; 32.7]	0.2
Comorbidities				
Cancer	14 (12)	9 (13)	5 (9.8)	0.55
Cardiovascular disease	77 (65)	42 (63)	35 (69)	0.5
Chronic skin disease	12 (10)	8 (12)	4 (7.8)	0.45
Diabetes	49 (42)	26 (39)	23 (45)	0.49
IVDU	15 (13)	8 (12)	7 (14)	0.77
Kidney disease	39 (33)	26 (39)	13 (25)	0.13
Liver disease	4 (3.4)	3 (4.5)	1 (2)	0.63
Respiratory disease	36 (31)	20 (30)	16 (31)	0.86
Immunosuppressive therapy	22 (19)	17 (25)	5 (9.8)	0.031
History of MRSA infection (<1y)	31 (26)	13 (19)	18 (35)	0.052
Bacteremia acquisition				
Community (CA)	8 (6.8)	1 (1.5)	7 (13.7)	0.02
Healthcare (HACO)	82 (69.5)	44 (65.7)	38 (74.5)	0.3
Nosocomial (HA)	28 (23.7)	22 (32.8)	6 (11.8)	<0.01
Bacteremia source				
Urinary	5 (4.2)	5 (7.5)	0 (0)	0.2
Respiratory	8 (6.8)	7 (10)	1 (2)	0.14
Surgical site	17 (14)	4S (6)	13 (25)	<0.01
Skin site	28 (24)	16 (24)	15 (29)	0.5
CVC infection	14 (12)	11 (16)	3 (5.9)	0.08
Unknown	37 (31)	21 (31)	18 (35)	0.65
Arteriovenous graft	4 (3.4)	3 (4.5)	1 (2)	0.63
Presence of >1 foreign body	33 (28)	13 (19)	20 (39)	0.018
Time between FB implantation and positive BC, months, med [Q25-75]	38.3 [7.9; 89.3]	48.3 [19.4; 92.6]	19 [4.7; 56.6]	0.013
Time <18 months	71/112 (63)	16/61 (26)	25/51 (49)	0.013
Days of positive BC, med [Q25-75]	3.00 [1.00; 7.00]	1.00 [1.00; 3.00]	7.00 [3.00; 11.0]	<0.01
Positive blood culture ≥48h	73 (62)	28 (42)	44 (86.3)	<0.01
Sofa score, med [Q25-75]	3.00 [1.00; 5.75]	4.00 [1.00; 7.00]	2.00 [1.00; 5.00]	0.19
Pitt bacteremia score, med [Q25-75]	1.00 [0; 2.00]	1.00 [0; 3.00]	1.00 [0; 2.00]	0.69
Native valve Infective endocarditis	6 (5.1)	3 (4.5)	3 (5.9)	1
Duration of antibiotic, med [Q25-75]	44.0 [28.0; 55.0]	42.0 [27.0; 45.0]	52.0 [43.5; 58.5]	<0.001
Duration of Hosp, med [Q25-75]	15.5 [10.0; 25.0]	15.0 [9.00; 22.5]	20.0 [12.5; 27.0]	0.041
In-hospital mortality	17 (14)	10 (15)	7 (14)	0.85
Phenotypic characteristics*				
Biofilm, med [Q25-75], OD 570 nm	0.405 [0.281; 0.602]	0.365 [0.272; 0.603]	0.445 [0.288; 0.594]	0.39
Biofilm (moderate/strong)	45 (39)	25 (38)	20 (40)	0.82
Molecular characteristics**				
ST8	45 (39)	23 (36)	22 (44)	0.38
ST5	33 (29)	21 (33)	12 (24)	0.3
ST105	23 (20)	14 (22)	9 (18)	0.61
Other STs †	13 (11)	6 (9.4)	7 (14)	0.44
PVL gene carriage	36 (32)	19 (30)	17 (34)	0.62

* No biofilm data (n=2).

** No sequencing data (n=4).

**†** ST1 (n=1), ST30 (n=1), ST59 (n=1), ST72 (n=1), ST87 (n=1), ST772 (n=1), ST840 (n=2), ST1472 (n=1), ST3059 (n=1), new ST (n=3).

BC, blood culture; CA, community-associated; CVC, central venous catheter; FB, foreign body; HA, healthcare-associated; HACO, healthcare-associated, community-onset; IVDU, intravenous drug use; med, median; PVL, Panton-Valentine leucocidin; ST, (multilocus) sequence type.

**Table 2:** Multivariate logistic regression model.

	Univariate p-value	Multivariable p-value	Multivariable OR (CI 95)
Race	0.041	NT	
Immunocompromised therapy	0.031	NT	
History of MRSA infection (<1y)	0.052	0.012	4.7 (1.4-15.5)
Nosocomial	<0.01	NT	
Community	0.02	0.003	68.1 (4.2-1114.3)
Source of infection: surgical site	<0.01	0.007	11.8 (2-70.4)
Source of infection: CVC	0.08	NT	
Presence of >1 foreign body	0.018	0.033	3.4 (1.1-10.7)
Time between FB implantation and positive BC <18 months	0.013	0.031	3.3 (1.1-10)
Positive BC ≥48h	<0.01	<0.001	16.7 (4.3-65.7)

All variables with p<0.1 in univariate analysis were included. A stepwise, backward-selection analysis was performed with an entry p=0.05 and suppression at p=0.1.

BC, blood culture; CVC, central venous catheter; FB, Foreign body; NT, not included in the final model.

### 3.3 Sequence type

**Figure 2:**
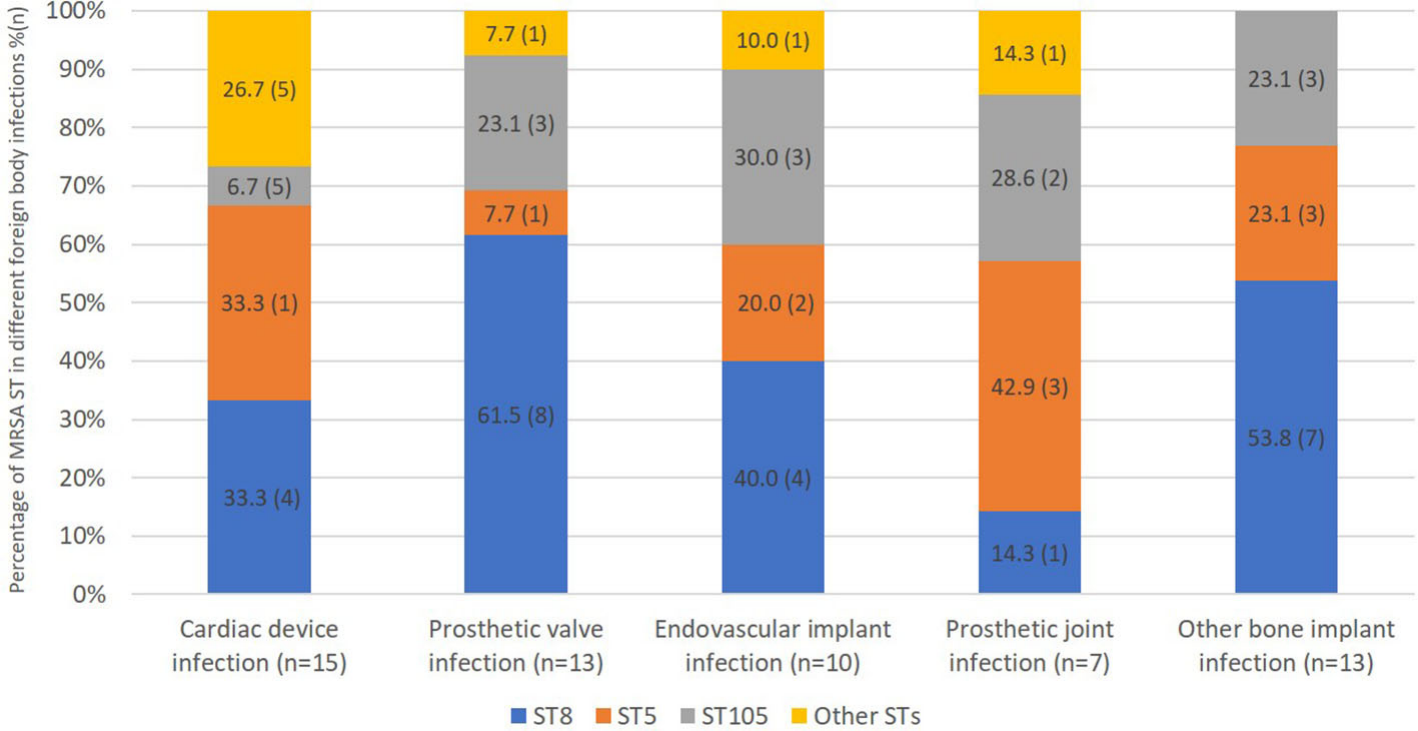
Distribution of MRSA sequence types according to foreign body infection type.

We were able to obtain whole genome sequence data for 114/118 isolates (**Table 1**). The most prevalent sequence type was ST8 (39%), followed by ST5 (29%), and ST105 (20%). There was no significant difference in ST comparing patients with or without foreign body infection (**Table 1**). Moreover, 36 isolates carried Panton Valentin leucocidin (PVL) genes, 34 in MRSA ST8, 1 in MRSA ST772 (CC1) and 1 in a novel, previously unassigned ST belonged to CC8. MRSA ST8 tended to be more frequent in patients with prosthetic valve infection than patients with PJI, and MRSA ST5 tended to be more frequent in patients with PJI than prosthetic valve infection (8/13, 62% vs. 1/7, 14%, p=0.07 and 3/7, 43% vs. 1/13, 7.7%, p=0.1, respectively) although these associations were not significant (**Figure 2**).

### 3.4 Biofilm formation

**Figure 3:**
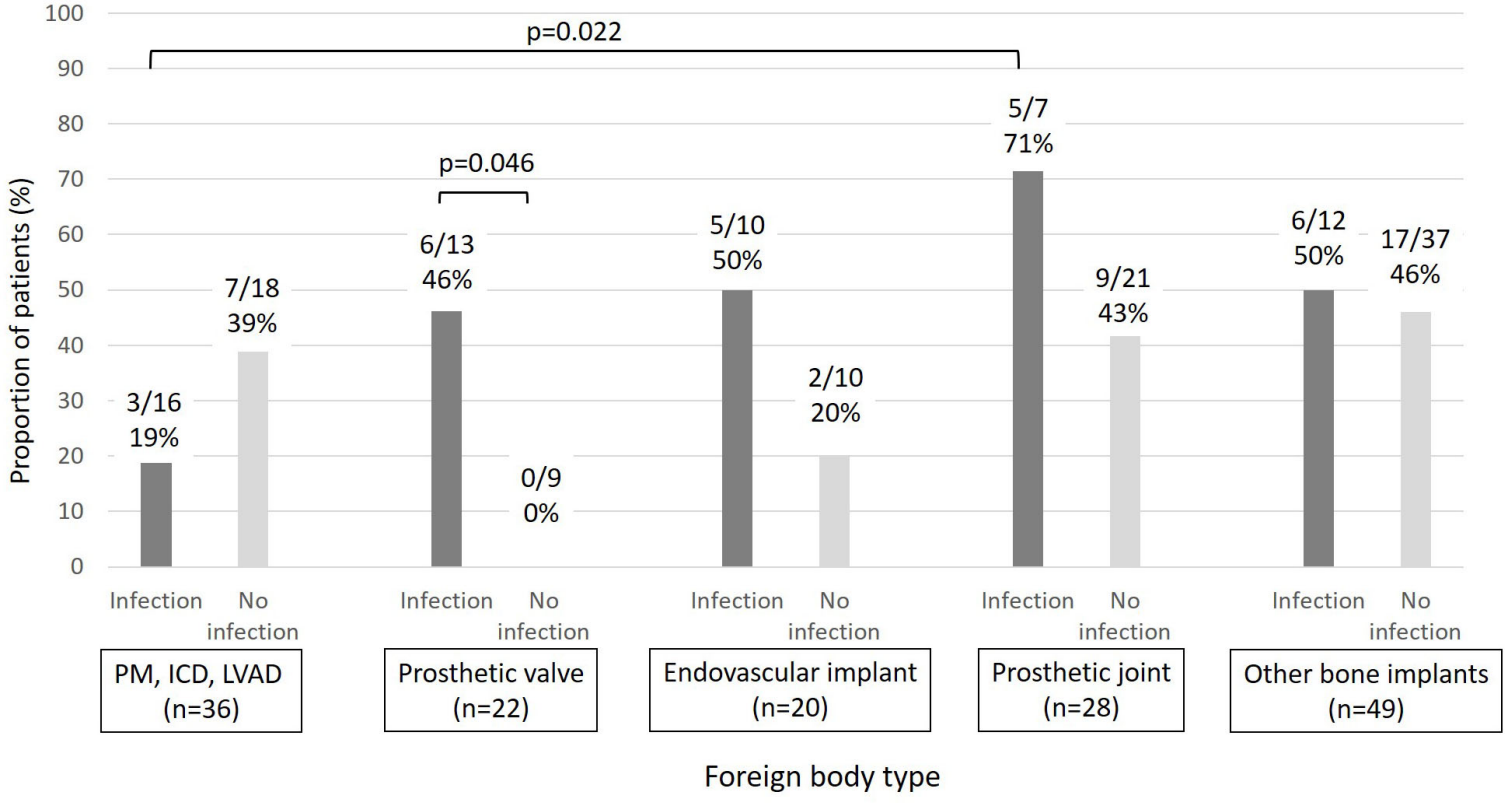
Proportion of patients with biofilm-forming MRSA (moderate/strong) by foreign body type and foreign body infection (PM: pacemaker, ICD: implantable cardiac defibrillators, LVAD: Left ventricular assistance devices).

*In vitro* biofilm formation assays were performed for 116/118 MRSA isolates (**Table 1**). Only 39% (45/116) of isolates formed a moderate/strong biofilm. No significant difference was observed in moderate/strong biofilm formation between patients with foreign body infection and those without foreign body infection (20/45, 44% vs. 30/71, 42%, p=0.82) (**Table 3**). Among subjects with a prosthetic heart valve (n=22), those with a prosthetic heart valve infection were more likely to have an isolate with moderate/severe biofilm production than subjects without prosthetic heart valve infection (6/13, 43% vs. 0/9, p=0.046). Moreover, MRSA BSI isolates in subjects with PJI produced more biofilm than those from subjects with a cardiac device infection (5/7, 71% vs. 3/16, 19%, p=0.022) (**Figure 3**). In multivariable analysis, subjects infected with a MRSA isolate producing moderate/strong *in vitro* biofilm were more likely to have a history of MRSA infection in the last year (OR=3.41 [1.23-9.43]), interval between foreign body implantation and MRSA BSI <18 months (OR=3.1 [1.05-9.2]), and ST8 (OR=10.64 [2-57.3]) (**Table 3**).

**Table 3:** Factors associated with MRSA producing in vitro moderate/strong biofilm.

Characteristics	Weak/No biofilm (n=71)	Moderate/strong biofilm (n=45)	p-value	Multivariable OR (IC 95%)	Multivariable p-value
Age, med [Q25-75]	61.4 [48.9; 69.6]	60.8 [50.7; 70.4]	0.66		
Female sex	31 (44)	15 (33)	0.27		
Race			0.38		
White	25 (35)	22 (49)			
Black	34 (48)	18 (40)			
Other	4 (5.6)	3 (6.7)			
Unknown	8 (11)	2 (4.4)			
Body mass index, med [Q25-75]	28.0 [24.0; 31.2]	26.7 [22.6; 32.9]	0.5		
Comorbidities					
Cancer	7 (9.9)	5 (11)	1		
Cardiovascular disease	49 (69)	26 (58)	0.22		
Chronic skin disease	9 (13)	3 (6.8)	0.37		
Diabetes	28 (39)	21 (47)	0.44		
IVDU	7 (9.9)	8 (18)	0.22		
Kidney disease	29 (41)	10 (22)	0.039	NT	
Liver disease	3 (4.2)	1 (2.2)	1		
Respiratory disease	23 (32)	12 (27)	0.51		
Immunosuppressive therapy	13 (18)	7 (16)	0.7		
History of MRSA infection (<1y)	10 (14)	19 (42)	<0.001	3.41 (1.23-9.43)	0.018
Bacteremia acquisition					
Community (CA)	5 (7)	3 (6.7)	1		
Healthcare (HACO)	44 (62)	36 (80)	0.041	NT	
Nosocomial (HA)	22 (31)	6 (13)	0.03		
Bacteremia source					
Urinary	1 (1.4)	4 (8.9)	0.074	NT	
Respiratory	7 (9.9)	1 (2.2)	0.15		
Surgical site	8 (11)	8 (18)	0.32		
Skin site	18 (25)	12 (27)	0.87		
CVC infection	9 (13)	5 (11)	0.8		
Unknown	25 (35)	14 (31)	0.65		
Arteriovenous graft	3 (4.2)	1 (2.2)	1		
Presence of >1 foreign body	22 (31)	11 (24)	0.45		
At least one foreign body infection	30 (42)	20 (44)	0.82		
Cardiac devices	13 (18)	3 (6.7)	0.076	0.23 (0.04-1.29)	0.097
Prosthetic heart valve	7 (9.9)	6 (13)	0.56		
Endovascular implants	5 (7)	5 (11)	0.51		
Prosthetic joint infection	2 (2.8)	5 (11)	0.11		
Other bone implants	6 (8.5)	6 (13)	0.53		
Time between FB implantation and positive BC <18 months	39 (57)	32 (76)	0.036	3.1 (1.05-9.2)	0.041
Positive blood culture ≥48h	43 (61)	29 (64)	0.67		
Sofa score, med [Q25-75]	4.00 [2.00; 6.25]	1.00 [0; 5.00]	<0.01	NT	
Pitt bacteremia score, med [Q25-75]	1.00 [0; 3.00]	0 [0; 2.00]	0.029	NT	
Duration of antibiotic, med [Q25-75]	43.0 [27.0; 50.5]	51.0 [42.0; 59.0]	0.019	NT	
Duration of Hosp, med [Q25-75]	15.0 [10.5; 24.0]	17.0 [11.0; 28.0]	0.76		
In-hospital mortality	13 (18)	4 (8.9)	0.16		
Molecular characteristics					
ST8	19 (28)	25 (57)	<0.01	10.64 (2-57.3)	0.006
ST5	23 (34)	10 (23)	0.21		
ST105	16 (24)	6 (14)	0.2		
Other STs*	10 (15)	3 (6.8)	0.2		
PVL carriage	18 (26)	18 (41)	0.11		

BC, blood culture; CA, community-associated; CVC, central venous catheter; FB, foreign body; HA, healthcare-associated; HACO, healthcare-associated, community-onset; IVDU, intravenous drug use; med, median; NT, not included in the final model; PVL, Panton-Valentine leucocidin; ST, (multilocus) sequence type.

## 4 Discussion

We studied clinical and microbiologic risk factors for foreign body infections complicating MRSA BSI in a cohort of 124 adult patients with a foreign body *in situ* at the time of BSI diagnosis. One-third of all 423 patients with a MRSA BSI had a foreign body, and 43% of these 124 subjects had a foreign body infection complicating the BSI. Patients with bone implants were rarely infected. *In vitro* level of biofilm formation by MRSA isolates was not associated with risk of foreign body infection, but high-level biofilm formation was more common in patients with a history of MRSA infection and MRSA ST8 infection.

Many studies have analyzed foreign body infection in patients with MRSA infection and the presence of at least one foreign body at the time of BSI (19–27). However few studies have analyzed the role of biofilm and genetic characteristics of MRSA in this population. Interestingly, in our study neither comorbidities nor demographic characteristics were associated with risk of foreign body infection. These results contrast with previously identified risk factors for metastatic infection in MRSA BSI, probably because only patients with a foreign body *in situ* were included in this study. In previous studies, CA-MRSA was found to be associated with metastatic infection and persistent bacteremia, as we found (19, 28). Moreover, persistence of positive blood cultures after initiation of antimicrobial therapy was identified as a risk factor for complicated bacteremia, cardiac device infection, and infective endocarditis, but also 90 day-mortality (19, 25, 29, 30).

The pathogenicity of *S. aureus* in foreign body infection is based on its ability to adhere, colonize, and persist on a foreign body. The impact of *S. aureus* biofilm on the clinical characteristics and outcome of foreign body infection has little been studied. Previous studies showed contradictory results on an association between *in vitro* biofilm production and clinical outcome. Alonso et al. analyzed patients with *S. aureus* bacteremia and infective endocarditis, catheter-related bacteremia or non-device associated bacteremia. No association was found between biofilm production (using a crystal violet assay), biofilm metabolic activity (using XTT solution) and the 3 different groups of infection (31). Mesrati et al. evaluated biofilm formation among *S. aureus* isolates responsible for foreign body infections. The foreign bodies included central venous catheters (n=24), indwelling urethral catheters (n=3), hemodialysis catheters (n=3), prosthetic joint (n=8), tracheal catheters (n=1), and nasogastric tubes (n=1). Among the 87 *S. aureus* isolates, 5 produced strong, 20 moderate and 35 weak biofilm. Biofilm production was statistically greater in the foreign body group compared with the non-foreign body group (80% vs. 60%; P=0.04). However, MRSA strains of *S. aureus* were reported in only 9/40 in the device group and 23/47 in the non-device group (32).

Although studies of *in vitro* biofilm production comparing *S. aureus* isolates from different types of infections have been contradictory, biofilm production has previously been associated with certain clonal lineages of *S. aureus*. Fernández-Hidalgo et al. analyzed 209 *S. aureus* strains causing infective endocarditis and found that strains belonging to CC5 and CC22 had greater biofilm formation, measured as significantly higher optical densities (1.369 [1.18] vs. 0.920 [0.93], p = 0.008). However, only 20% of isolates were MRSA, and CC5 was the most prevalent (22%) followed by CC30 (19%) and CC8 (11%). Similarly, Naicker et al. showed that in 30 genotypically varied strains isolated from blood culture, only 5 isolates were strong biofilm producers, and all belonged to one *spa* type clonal cluster (*spa*-CC 064) related to CC5 and CC8 (33).

The results of our study may be impacted by the molecular epidemiology of MRSA at the medical center studied. However, the distribution of STs in our study was similar to previous studies on MRSA infections in the U.S. MRSA ST5 and ST105 belonging to CC5 were reported to be the most prevalent CC in MRSA infection in the US (69%), followed by CC8 and CC30 (28). In our study, only one ST30 isolate was found. The specific ST8 MRSA clone USA300 commonly carries the PVL and the Arginine Catabolic Mobile Element (ACME). It is likely that most of the ST8 isolates were USA300. In MRSA BSI, the USA300 lineage has been associated with a higher risk of metastatic infection, emboli, and persistent bacteremia (28). USA300 has been shown to be an excellent biofilm former in two biofilm models. In an *in vitro* study of 76 MRSA isolates classified into 13 clones, static biofilm (microtiter plate) and dynamic biofilm with confocal laser-scanning and time-lapse microscopy were performed. In the two models, the USA300 clone formed more biofilm than others. In fact, strains carrying SCC*mec* type IV generally showed greater biofilm production than those carrying other SCC*mec* (I-III) types. Thus, both SCC*mec* type IV and ACME carriage seem to be associated with increased biofilm formation (34), although there is very likely confounding in the relationship between SCC*mec* type and ST and there may not be an independent association with SCC*mec* type. In any case, it is likely that differences in genotype are a critical factor in observed variation in biofilm formation.

Methods used to evaluate *in vitro* biofilm formation in *S. aureus* have differed among published studies, and this may have affected observed study results in the literature. Whereas Christensen et al. was one of first authors to describe the microtiter plate biofilm method in coagulase negative staphylococci, Stepanovic et al. reported a more precise protocol, and explained the variability of such different protocols (17, 35). The main elements of biofilm production assay protocols that Stepanovic et al. standardized and that we utilized were 1) a well-defined concentration of bacteria (0.5 McFarland) before filling the 96-well plate, 2) the use of tryptic soy broth (TSB) or brain-heart infusion (BHI) with supplementation of 1% glucose to increase biofilm formation, 3) rinsing the plate at least 3 times after bacterial culture, and 4) the method used to calculate the ODc, using the negative control (17). Furthermore, our experiment were performed by a single person, which limited inter-variability, especially when it came to rinsing the plate with the same pressure. Although we applied the protocol of Stepanovic et al., we did not demonstrate an association between MRSA biofilm formation and foreign body infection.

There are certain limitations to our study. First, we had a relatively small sample size and included different types of foreign bodies, with distinct pathophysiology of infection. Thus, we may not have had adequate power to demonstrate a significant association between level of biofilm formation and risk of foreign body infection. Future studies including only one type of foreign body in MRSA BSI patients may show different results. Second, *S. aureus* biofilm formation in foreign body infection may not be replicated by the microtiter plate biofilm assay. Study of biofilm formation *in vitro* or *in vivo* on a relevant tissue and/or a foreign body surface more similar to those implanted in patients may be warranted to better evaluate the role of biofilm in foreign body infection. Third, it is possible that there were BSI subjects in our study with a prosthetic joint and no local symptoms who had a missed diagnosis of a true PJI. However, our patients were followed up after BSI to avoid this misclassification. Moreover, the absence of clinical symptoms in patients with an orthopedic device and a *S. aureus* BSI usually rules out a PJI (23). In a study by Dufour et al., among patients with *S. aureus* BSI and a prosthetic joint 27/143 (19%) had at least one PJI, and for all PJI except one, diagnosis was made during the same hospitalization as MRSA BSI (20).

## 5 Conclusion

Most factors associated with foreign body infection in MRSA BSI were also characteristic of persistent infections. Patients with bone implants were rarely infected. Biofilm-forming isolates were not associated with a higher risk of foreign-body infection. However, high-level *in vitro* biofilm formation was associated with MRSA genetic lineage, especially ST8. Animal studies may be needed to confirm that *in vitro* biofilm formation corresponds to *in vivo* biofilm formation and is not simply a lineage-specific marker of MRSA *in vitro*.

## Data availability statement

The original contributions presented in the study are publicly available. This data can be found here: Sequence Read Archive (Bioproject PRJNA751847).

## Ethics statement

The studies involving humans were approved by the Institutional Review Board of the University of Pennsylvania. The studies were conducted in accordance with the local legislation and institutional requirements. Written informed consent for participation was not required from the participants or the participants’ legal guardians/next of kin because this study was exempt from the need for informed consent by the Institutional Review Board of the University of Pennsylvania.

## Author contributions

KB: Conceptualization, Data curation, Formal analysis, Funding acquisition, Investigation, Methodology, Writing – original draft. NJ: Investigation, Project administration, Writing – review & editing. MS: Investigation, Writing – review & editing. BT: Formal analysis, Writing – review & editing. TR: Formal analysis, Writing – review & editing. MD: Conceptualization, Funding acquisition, Methodology, Resources, Supervision, Writing – review & editing.

## Supplementary material

The supplementary file contains a more detailed section of the method.
