# Supplementary material for: Factors associated with foreign body infection in methicillin-resistant Staphylococcus aureus bacteremia
Source: Front Immunol. 2024 Feb 16;15:1335867. doi: 10.3389/fimmu.2024.1335867 (PMC10904584; doi:10.3389/fimmu.2024.1335867)
Supplement: Supplementary file 1 [file DataSheet_1.docx]

Supplementary Material

# *In vitro* mature biofilm formation

Prior to inoculation, all strains were transferred from the frozen stock cultures to trypticase soy agar (TSA) and incubated aerobically at 37°C overnight. Then, all strains were subcultured once more under the same conditions. Organisms isolated from fresh agar plates were inoculated in 5 mL of standard trypticase soy broth (TSB) and vortexed for one minute. Broth samples were incubated in the orbital shaker incubator at 37°C, 200 rpm, for 18 h +/-30min. After vortexing, the bacterial suspension was standardized to an optical density at 600 nm of 1.00 ± 0.05 (0.5 McF). The cultures were then diluted 1:100 in TSB supplemented with 1% glucose. Sterile flat-bottomed 96-well polystyrene tissue culture-treated microtiter plates were filled with 200 μL of the diluted cultures. The negative control wells contain broth only. The positive control (MRSABB_04026**)**, a strong biofilm-forming strain, was selected. The plates were incubated aerobically at 37°C for 24 h under static conditions. After incubation, the contents of each well were removed by gentle tapping. The wells were washed with 200μl of phosphate buffered saline (PBS, pH 7.2) three times using an appropriate micropipette. Following every washing step, the wells were emptied by flicking the plates. Prior to fixation of the biofilm, the plates were drained in an inverted position (at least 20 min). The biofilm was fixed with 150μl of methanol for 20 min. Next, the microtiter plates were emptied by simple flicking, and left to air dry for at least 3 hours in an inverted position at room temperature. Then, 150μl of 0.1% crystal violet solution was added to each well containing dry biofilm for 15 min at room temperature. After staining, the stain was aspirated with a pipette and excess stain was rinsed off by placing the microtiter plate under running tap water. The microplate was air dried at room temperature (at least 20 minutes). The cells were resolubilized, eluted from attached cells with 150 μl of 95% ethanol per well for at least 30 min without shaking. Optical density (OD) of stained adherent biofilm was obtained by using an automated microtiter plate reader (SpectroStar Nano spectrophotometer, BMG Labtech, Germany) at wavelength 570 nm. Each experiment was performed in triplicate technical replicates, and each experiment was repeated three times.

**Results**


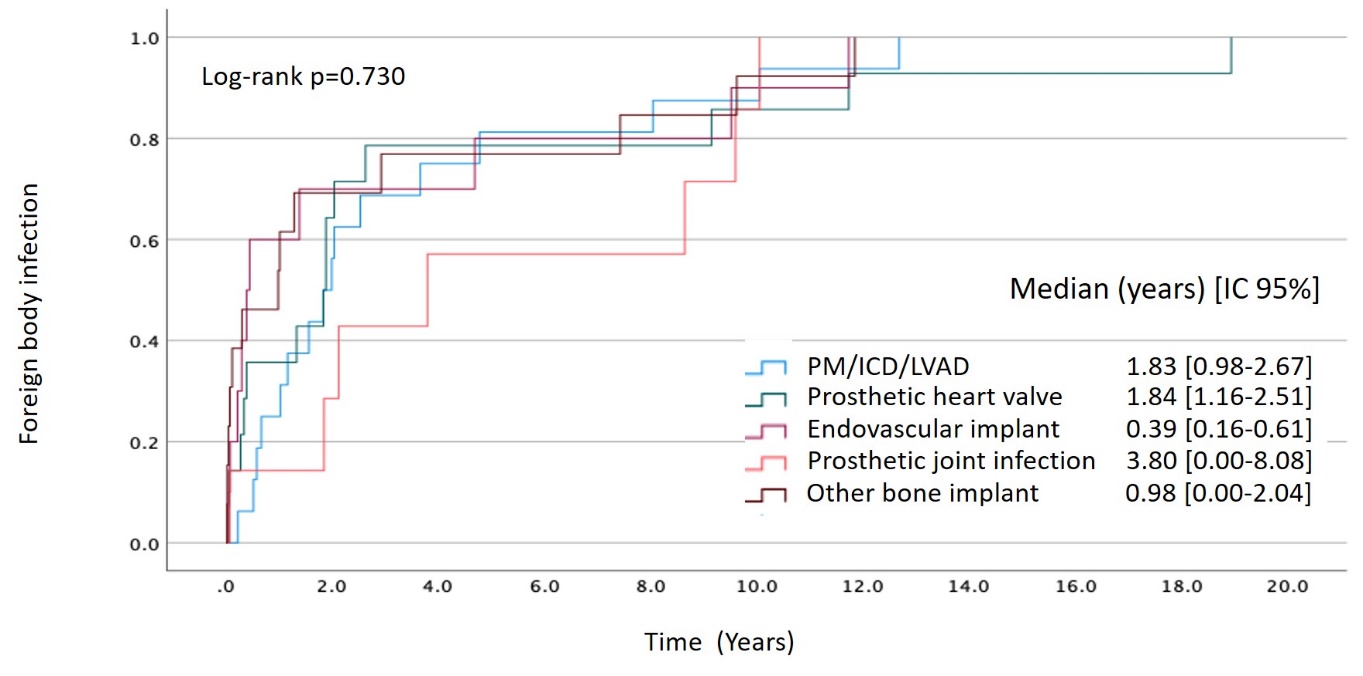


**Figure S1**: Time Between Date of Foreign Body Implantation and MRSA Foreign Body Infection, Years. PM: pacemaker, ICD: intra cardiac defibrillator, LVAD, left ventricular assistant device
